# Microbiota analysis of peri-implant mucositis in patients with periodontitis history

**DOI:** 10.1007/s00784-022-04571-1

**Published:** 2022-06-08

**Authors:** Na Zhou, Haohao Huang, Hui Liu, Qiang Li, Guangwen Yang, Yu Zhang, Meng Ding, Heng Dong, Yongbin Mou

**Affiliations:** 1 Department of Jiangbei, Nanjing Stomatological Hospital, Medical School of Nanjing University, Nanjing, China; 2 Department of Oral and Maxillofacial Surgery, Nanjing Stomatological Hospital, Medical School of Nanjing University, Nanjing, China; 3 Department of Oral Implantology, Nanjing Stomatological Hospital, Medical School of Nanjing University, Nanjing, China

**Keywords:** Microbiota, Periodontitis, Dental implants, Mucositis, Dental plaque

## Abstract

**Objectives:**

To investigate the bacterial diversity in peri-implant plaques and the effect of periodontitis history on the occurrence of peri-implant mucositis.

**Materials and methods:**

Three groups of subgingival plaques were collected from peri-implant sulci in the first molar area. The three groups included healthy implants in patients without periodontitis (NH implant), healthy implants in patients with periodontitis history (PH implant), and peri-implant mucositis implants in patients with periodontitis history (PM implant). Subgingival plaques in periodontal pockets of contralateral natural first molars were also collected. Bacterial DNA was extracted and the V4 region of the 16S rDNA sequence was amplified and sequenced on an Illumina HiSeq platform. The operational taxonomic units obtained from amplicon sequencing were used to analyze the prevalence and identity of bacteria based on public databases and advanced techniques.

**Results:**

Analysis of similarities indicated a significant difference in bacterial structures between the NH implant and PM implant groups. Additionally, a significantly higher relative abundance of the genera *Actinomyces* and *Streptococcus* was found in the samples of the NH implant group. The genera *Fusobacterium* and *Prevotella* could be considered as potential biomarkers for peri-implant mucositis. Moreover, more gram-negative anaerobic bacteria (*Porphyromonas* and *Prevotella*) were detected in the samples from patients with periodontitis history.

**Conclusions:**

The increased accumulation of *Fusobacterium* and *Prevotella* is associated with a higher risk of peri-implant mucositis. In addition, patients with periodontal history may be more likely to develop peri-implant mucositis.

**Clinical relevance:**

The increase in periodontal pathogens and the decrease in health-associated bacteria in patients with periodontitis history may be more likely to develop peri-implant mucositis. These results provide a bacteriological basis for the prevention and treatment of peri-implant mucositis in patients with periodontitis history.

**Supplementary Information:**

The online version contains supplementary material available at 10.1007/s00784-022-04571-1.

## Introduction

Dental implant treatment can restore missing teeth with a reliable prognosis, but the high prevalence of peri-implant diseases has become an increasing problem that threatens the long-term stability and satisfaction of clinical outcomes [1–5]. Notably, the occurrence of peri-implant diseases was closely related to microbial colonization and host response, which was in accordance with periodontal diseases [6, 7]. Peri-implant mucositis, considered the precursor for peri-implantitis, is one of the most common complications caused by dysbiosis of the peri-implant microenvironment at the implant-mucosa interface. Nearly 23.9–88.0% of patients and 9.7–81.0% of implants suffered from peri-implant mucositis [8–11]. Therefore, the etiological factors of peri-implant mucositis are worth exploring.

Oral disease pathology is attributed not only to key pathogens but also to networks of co-occurring microbes [12]. The human oral microbiome mainly exists on the external surface of teeth or prostheses growing as a complex biofilm ecosystem, named dental plaque [13–15]. Because of pathogenic bacteria and interactions with the immune defenses of the host, dental bacterial plaque has been considered the initial factor of periodontal diseases [16]. Whether bacterial diversity could further result in peri-implant mucositis and even increase the probability of peri-implantitis is of scientific relevance.

Periodontitis affects the microbial composition of dental plaque and has been suggested to be a risk factor for peri-implantitis and implant failure [17–19]. Although periodontitis can be controlled after treatment, there are still compromised sites at the time of implant installation [20]. The periodontal pocket is regarded as a natural microbial reservoir, and the translocation of periodontal pathogens from the remaining dentition to peri-implant sites is an indispensable factor in the etiology of peri-implant disease [21, 22]. Thus, we hypothesized that the microbiome of peri-implant sites might be different between healthy individuals and patients with periodontitis history, even though all their implants had healthy status. A recent study demonstrated that peri-implantitis and healthy sites in periodontitis patients appeared to have unique microbiological ecosystems, but the microbiome of peri-implant mucositis sites was overlooked or confounded with peri-implantitis [23, 24]. Although some authors have demonstrated that the microbial communities of peri-implant mucositis sites were intermediate between peri-implantitis sites and healthy implants, the influence of periodontitis history was not considered [25].

In the present study, we identified the bacterial diversity in peri-implant plaques of different patients to further explore the relationships between periodontitis history and the occurrence of peri-implant mucositis. These findings in microbiology might lead to the development of new opinions about the etiology of peri-implant mucositis and help us to prevent and manage inflammation at an early stage of peri-implant disease in the future.

## Materials and methods

### Recruitment of participants

Patients were voluntarily recruited at Nanjing Stomatological Hospital, Medical School of Nanjing University from May 2018 to November 2018. The inclusion criteria were as follows: (1) patients who completed the final implant restoration at the first molar area for more than 6 months, (2) patients who had only one bone-level dental implant, (3) patients who had good compliance and received oral health education, and (4) patients who agreed and signed the informed consent form. The exclusion criteria were as follows: (1) smokers, (2) pregnant women, (3) patients with periodontitis in stage IV [26], (4) patients with severe implant infection (including peri-implantitis), (5) patients with antibiotics or steroid medications in the last 3 weeks, and (6) patients with uncontrolled systemic diseases (hypertension with blood pressure higher than 180/100 mmHg, diabetes mellitus with blood glucose higher than 8.88 mmol/L) or mental diseases (such as depression and mania).

The clinical characteristics of each patient were recorded, including sex, age, plaque index (PI), bleeding on probing (BOP), and peri-implant probing depth (PD). BOP and peri-implant PD were measured at six sites around each implant with a periodontal probe. Clinically, peri-implant mucositis is usually diagnosed by clinical inflammation signs, such as bleeding on gentle probing and visual signs of inflammation without marginal bone loss [27–29].

All the patients underwent panoramic radiographs before the surgical scheme design, and those with interproximal bone loss in more than 30% of the remaining teeth were assigned to the periodontitis history group in our study [26]. For the patients without obvious bone resorption, we further used a periodontal probe to check the periodontal attachment level. Patients without attachment loss were included in the group without periodontitis history. Finally, 33 participants were included in the present study. Eight patients had no periodontitis history, and the others had periodontitis history. Two independent dentists (NZ and HL) evaluated the clinical parameters. If they failed to reach an agreement, HD was the third one to make judgments. A representative image of peri-implant mucositis is shown in Figure S1.

### Microbial sampling collected

Plaque samples were collected from the bottom of the subgingival crevice using a sterile dental curette to avoid microbial DNA contamination according to previous studies [18, 19], and species were detected in each sample based on the 16S rRNA gene sequencing technique for identifying unidentified bacteria and providing reference identifications for unusual strains.

Prior to sampling, the implants of the included participants were examined, and a healthy implant was characterized by the absence of redness, swelling, bleeding on gentle probing, and suppuration. Individuals who exhibited inflammatory signs without progressive loss of supporting peri-implant bone were considered to have peri-implant mucositis implants. Thereafter, the samples obtained from the dental implants were divided into three analytical groups: healthy implants in patients without periodontitis (NH implant) group, healthy implants in patients with periodontitis history (PH implant) group, and peri-implant mucositis implants in patients with periodontitis history (PM implant) group. Representative X-ray images are shown in Figure S2.

To understand the differences in bacterial composition between natural teeth and implants (Figure S3), the subgingival plaques around the first molars were also collected in the same way simultaneously in each patient and were categorized into the NH tooth group (tooth in NH implant group), PH tooth group (tooth in PH implant group), and PM tooth group (tooth in PM implant group). All the samples were collected by Gracey curettes, immediately placed in 1.5 mL Eppendorf (EP) tubes containing 150 μL TE buffer (10 mM Tris–HCl, 1 mM EDTA, pH 7.6) and frozen at − 80 °C before DNA extraction.

### PCR amplification of the 16S rDNA gene

Bacterial nucleic acids were isolated using the Takara MiniBEST Bacteria Genomic DNA Extraction Kit (Takara, Japan) according to the manufacturer’s protocol, and bacterial DNA extraction was verified by agarose gel electrophoresis.

The V4 hypervariable region of the 16S ribosomal RNA (rRNA) gene was amplified by polymerase chain reaction (PCR) using a specific primer (515F-806R) with a specific barcode. All PCR runs in the present research were conducted with Phusion® High-Fidelity PCR Master Mix (New England Biolabs). Furthermore, the PCR products were mixed with equal volumes of loading buffer (1 × , containing SYBR green) and then electrophoretically separated on a 2% agarose gel. Only the samples with bright main strip bands between 400 and 450 bp could be used for further experimental study. Subsequently, PCR products were further purified using the QIAquick Gel extraction kit (Qiagen, Hilden, Germany) according to the manufacturer’s instructions.

### Sequencing and bioinformatics analysis

According to the instructions, sequencing libraries were generated using a DNA PCR-Free Sample Preparation Kit (TruSeq®, Illumina, USA), and index codes were added. Then, the quality of the library was evaluated by a Qubit 2.0 Fluorometer (Thermo Scientific) and Agilent Bioanalyzer 2100 system. The library was sequenced on an Illumina HiSeq 2500. Finally, 250 bp paired-end reads (PERs) were generated.

These PERs were assigned to each sample according to a unique barcode and truncated by cutting off the barcode and primer sequence. After the initial trimming, PERs were merged into longer reads by FLASH (V1.2.7), and the splicing sequences were named raw tags (RTs). Subsequently, quality filtering of the RTs was performed under specific filtering conditions to obtain high-quality clean tags (CTs) according to the QIIEM (V1.7.0). Then, the chimeric sequences that were detected by aligning the CTs with the data in the Gold database using the UCHIME algorithm were removed to finally create the effective tags.

Sequence analyses were performed by UPARSE software (UPARSE v7.0.1001, Edgar. 2013). With an identity threshold of 97% similarity, these sequences were assigned to the same operational taxonomic units (OTUs). The representative sequences of each OTU were annotated by the Ribosomal Database Project (RDP) classifier (Version 2.2). Then, the abundance of OTUs was normalized using a standard sequence number corresponding to the sample with the fewest sequences, and rarefaction curves, species accumulation boxplots and rank abundance curves were all generated using R software (Version 2.15.3). A heat map was generated by clustering analysis using the MRheatmap function. Based on the output normalized data, alpha diversity, including the analysis of richness estimators (Chao 1 and Ace) and diversity estimators (Shannon and Simpson), was analyzed using QIIME (Version 1.7.0). Additionally, beta diversity representing the diversity of the microbial community between sampling locations, was quantitatively calculated by the Bray–Curtis algorithm.

### Ethical approval

This study was approved by the institutional review board (IRB) of Nanjing Stomatological Hospital. The profiles of patients were obtained from the Department of Implantology and the Department of Information of Nanjing Stomatological Hospital and scrutinized by a disciplined investigator from Nanjing University. This study is in accordance with the Declaration of Helsinki.

### Statistical analysis

The UniFrac distances, showing the differences in bacterial compositions between the two samples, were calculated by QIIME software. R and P values were calculated to estimate the significance of intergroup differences, regarded as analysis of similarities (ANOSIM). Welch’s *t*-test and linear discriminant analysis (LDA) effect size (LEfSe) were performed to determine the potential biomarkers between each pair of groups. Significant differences were confirmed by values of *P* < 0.05.

## Results

### Basic information and overall microbial sequencing results

Thirty-three subgingival plaques collected around single-crown dental implants were included in the final analysis according to the inclusion and exclusion criteria. The subject demographics and clinical characteristics of the samples are listed in Table 1. Based on the Illumina HiSeq 2500 platform, 5,129,344 effective tags (mean/sample: 77,717) from a total of 5,173,398 raw tags were assigned to OTUs, and the average length was 253 bp. A complete list of observed OTUs is presented in Table S1. A species accumulation boxplot showed that each group had relatively adequate samples, as the newly observed species did not increase rapidly with increasing sample number (Figure S4A-D). Rarefaction curves directly reflecting the rationality of sequencing data size (Figure S4E) tended to level off, indicating that each sample in the final analysis had rational sequencing data. Furthermore, each sample had more than 250 observed species, and the gradual descending rank abundance curves indicated that the species were distributed evenly in all samples (Figure S4F). Thus, these thirty-three samples were used to further analyze the discrepancies in bacterial distribution among samples in groups.

**Table 1:** Subject demographics and clinical characteristics of the sampling sites

Parameters	NH implant (*n* = 8)	PH implant (*n* = 13)	PM implant (*n* = 12)	Total
Age (year ± SD)	39.25 ± 13.60	53.08 ± 4.66	55.67 ± 7.36	50.67 ± 10.56
Peri-implant PD (mm ± SD)	2.25 ± 0.46	2.85 ± 0.90	4.00 ± 1.04	3.12 ± 1.11
PI (mean ± SD)	0.75 ± 0.46	1.31 ± 0.48	2.00 ± 0.74	1.42 ± 0.75
BOP ( +)	0	0	12	12
Implant location (maxilla/mandible)	5/3	8/5	6/6	19/14

*PD*, probing depth; *PI*, plaque index; *BOP*, bleeding on probing

### Microbial diversity of each sample and the effect of periodontitis history

**Fig. 1:**
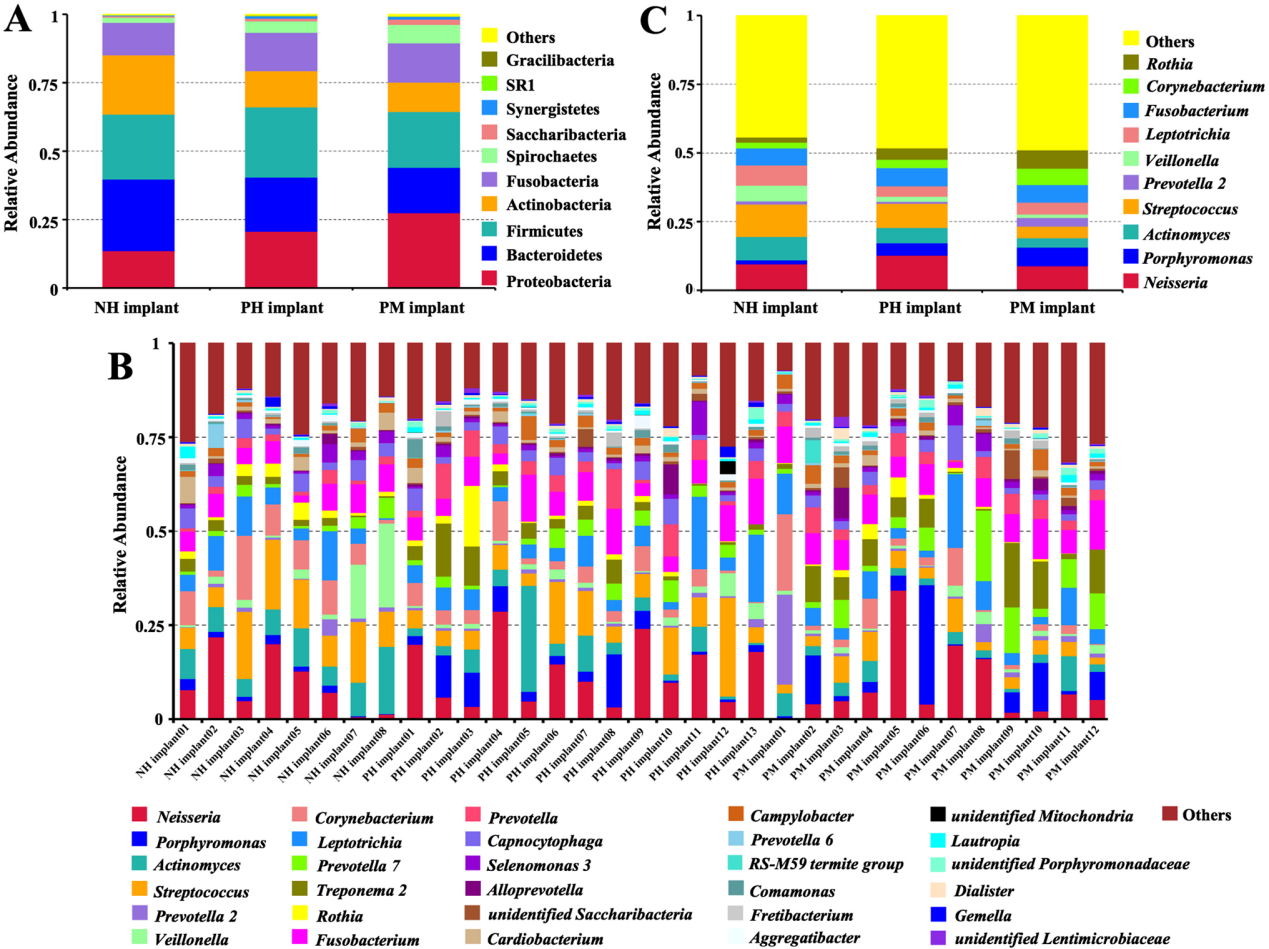
The relative abundance of major taxa and compositions of the microbiome. **A** Relative abundance of the top ten dominant phylotypes in the three groups. **B** Relative abundance of the top thirty genera for each sample. Each bar represents the relative abundance of each sample, and each color represents a particular bacterial genus. **C** Relative abundance of the top ten dominant genotypes

**Fig. 2:**
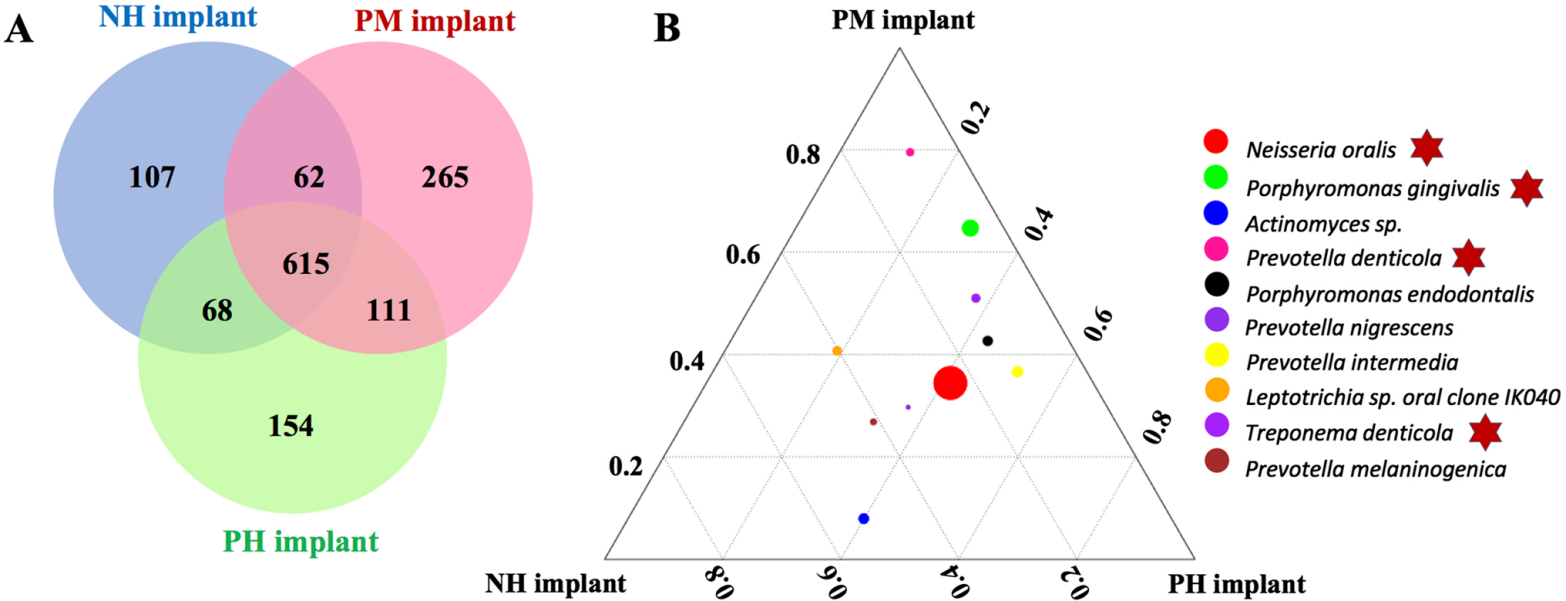
Similarity and differences among the three groups. **A** Venn diagram of the microbiome differences between peri-implant sites. OTUs in the overlapping regions were shared by two or three groups. **B** Ternary diagram of the bacterial species. The diameter of the circle indicates the abundance of each species

The relative abundance values of the top ten phylotypes (comprising 99.40–99.77% of the total counts) and genotypes (comprising 47.64–59.74% of the total counts) in each group are shown in Table 2. Based on the relative abundance, the composition of primary phyla and genera for each group were similar, but there were different distribution patterns (Fig. 1). Samples collected from patients with periodontitis history mainly harbored the phyla *Proteobacteria* and *Spirochetes*, whereas healthy individuals mainly harbored taxa from *Bacteroidetes* and *Actinobacteria* (Fig. 1A). The top 30 genera included approximately 75% of all taxa (Fig. 1B), and the top ten genera included almost 50% of all taxa (Fig. 1C). The distribution of each genus in samples in the PM implant group was more uniform. Then, the bacterial compositions around the implants were further evaluated by clustering analysis. Generally, samples in the three groups shared 615 OTUs, and 107 OTUs were unique in samples in the NH implant group, which was fewer than that of samples in the groups with periodontitis history. Moreover, samples in the PH implant and PM implant groups shared 111 OTUs, while samples in the NH implant group shared only 68 and 62 OTUs with samples in the PH implant and PM implant groups, respectively (Fig. 2A). A ternary diagram (Fig. 2B) showed the distribution trend of bacterial species with high abundance in the three groups. *Neisseria oralis* was abundant in all groups, and periodontal pathogens including *Porphyromonas gingivalis*, *Prevotella denticola*, and *Treponema denticola* were more closely related to the samples in the PM implant group.

**Table 2:** The relative abundances for the top 10 phyla and genera (%)

Taxonomy	NH implant	PH implant	PM implant
Phylum			
Bacteroidetes	13.6	20.7	27.5
Firmicutes	26.2	19.8	16.5
Proteobacteria	23.8	25.6	20.4
Actinobacteria	21.7	13.2	10.7
Fusobacteria	11.9	14.0	14.3
Spirochetes	1.9	4.2	6.8
Saccharibacteria (TM7)	0.5	0.9	1.9
Synergistetes	0.3	0.9	1.1
SR1	0.04	0.2	0.09
Cyanobacteria	0.01	0.09	0.01
Genus			
*Neisseria*	9.6	12.7	8.9
*Porphyromonas*	1.5	4.6	6.7
*Actinomyces*	8.5	5.6	3.4
*Streptococcus*	11.8	8.7	4.2
*Prevotella 2*	1.1	0.8	3.1
*Veillonella*	5.7	1.8	1.4
*Corynebacterium*	7.4	3.8	4.3
*Leptotrichia*	6.1	6.7	6.4
*Prevotella 7*	2.1	3.0	5.9
*Treponema 2*	1.9	4.2	6.7

A heat map generated by the supervised clustering algorithm is plotted in Figure S5, which shows the differences among samples in groups more quantitatively. Notably, the levels of periodontal pathogens such as *Porphyromonas*, *Prevotella*, *Treponema*, and *Leptotricia* (underlined by orange lines) were higher in samples from periodontitis patients (PH implant and PM implant groups). In contrast, healthy-related genera such as *Veillonella*, *Actinomyces*, and *Corynebacterium* (underlined by gray lines) were relatively higher in patients without periodontitis. In addition, infrequent taxa such as *Megasphaera*, *Parvimonas*, and *Dialister* were found to be clustered in samples from periodontitis patients, while *Selenomonas 3*, *Capnocytophaga*, and *Cardiobacterium* were clustered in samples from patients without periodontitis.

### Comparison of microbial profiles in samples in the three groups

**Fig. 3:**
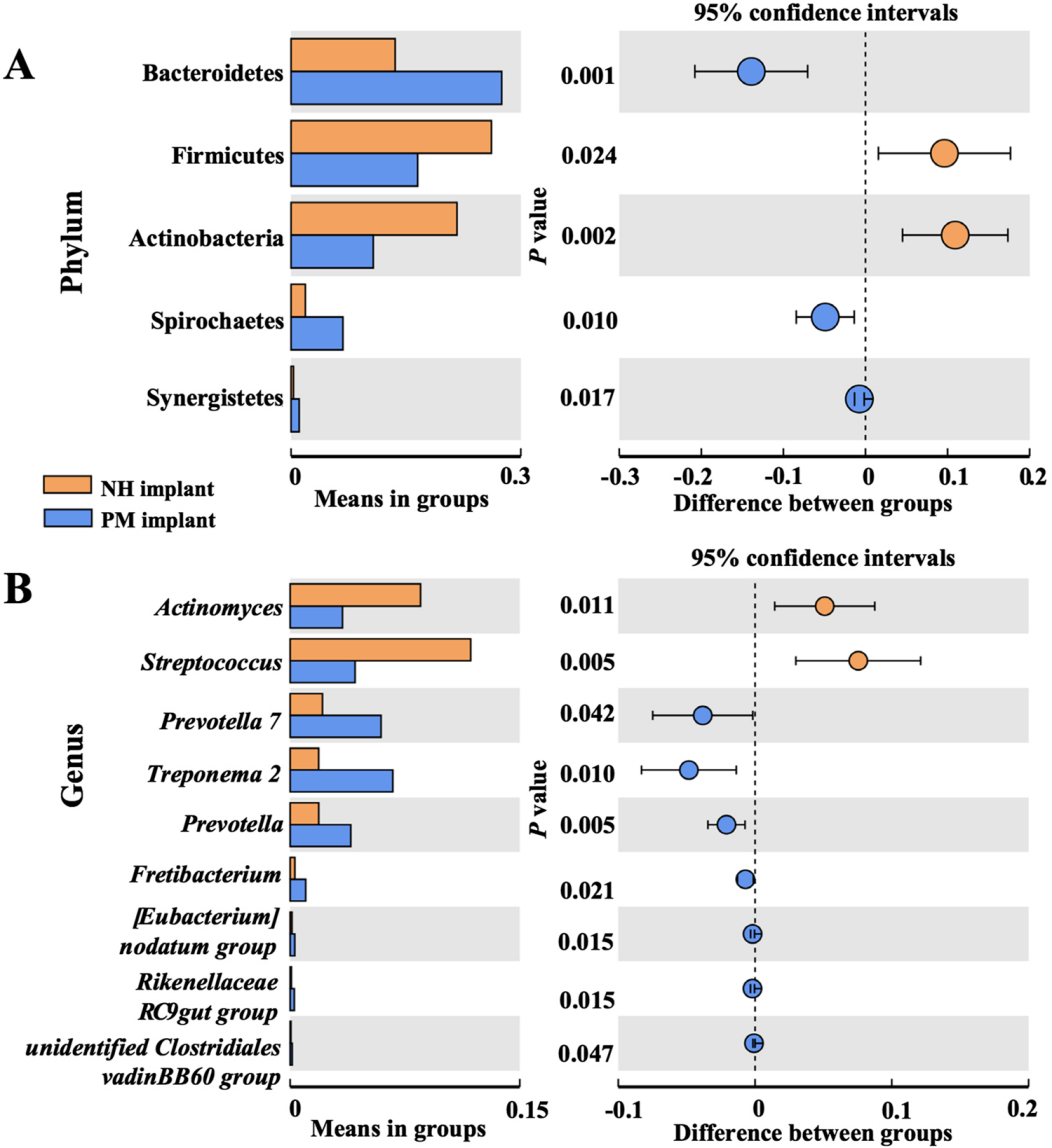
Relative microbial abundance at the phylum (**A**) and genus (**B**) levels between the NH implant and PM implant groups, which were analyzed by Welch’s *t*-test. Only taxa that were significantly different between groups were plotted (*P* < 0.05)

**Fig. 4:**
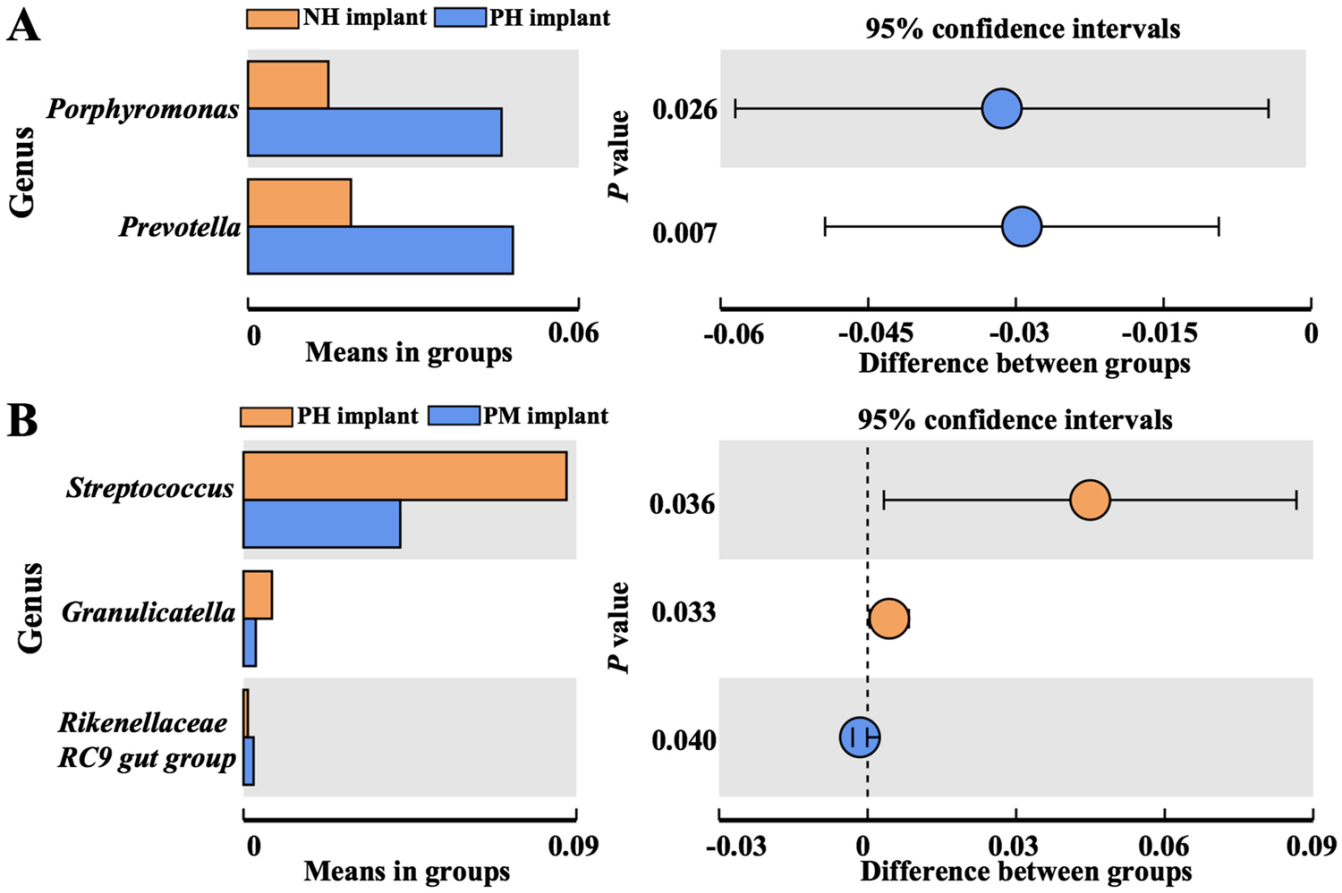
Relative microbial abundance at the genus level between groups analyzed by Welch’s *t*-test. **A** Differential bacterial genera between the NH implant and PH implant groups. **B** Differential bacterial genera between the PH implant and PM implant groups. Only taxa that were significantly different between groups were plotted (*P* < 0.05)

Alpha diversity analysis was conducted to analyze the statistical parameters, including observed OTUs, Shannon, Chao 1 index, and phylogenetic distance. The diversity and richness were similar among samples in groups (Figure S6). Analysis of similarity (ANOSIM) in beta diversity analysis showed significant differences in microbial community structures between samples in the two groups according to Bray–Curtis values (Table 3). The microbial community structures were significantly different between samples in the NH implant and PM implant groups (*P* < 0.05). Then, Welch’s *t*-test was conducted to identify specific phylum and genus between these two groups. The differential phyla (Fig. 3A) and genera (Fig. 3B) with significant differences between the NH implant and PM implant groups are listed. The levels of the genera *Actinomyces* and *Streptococcus* were higher in samples in the NH implant group, while *Prevotella 7*, *Treponema 2*, *Prevotella*, *Fretibacterium*, *[Eubacterium] nodatum* group, *Rikenellaceae RC9* gut group, and unidentified *Clostridiales vadin BB60* group were higher in samples in the PM implant group. Similar analysis was also carried out in samples in the other two groups (Fig. 4A, B), demonstrating that the levels of the genera *Porphyromonas* and *Prevotella* belonging to the phylum *Bacteroidetes* were higher in samples in the PH implant group than in samples in the NH implant group. Additionally, the levels of the genera *Streptococcus*, *Granulicatella*, and *Rikenellaceae RC9* gut groups were significantly different between samples in the PH implant and PM implant groups.

**Table 3:** Analysis of similarities based on Bray–Curtis values (within- vs. between-group rank dissimilarities)

Group	*R* value	*P* value
NH implant vs. PH implant	0.01	0.442
NH implant vs. PM implant	0.18	0.026*
PH implant vs. PM implant	0.05	0.124

*R* > 0, the sampling variation of the difference between two groups is greater than that within groups. *P < 0.05, the difference is significant

**Fig. 5:**
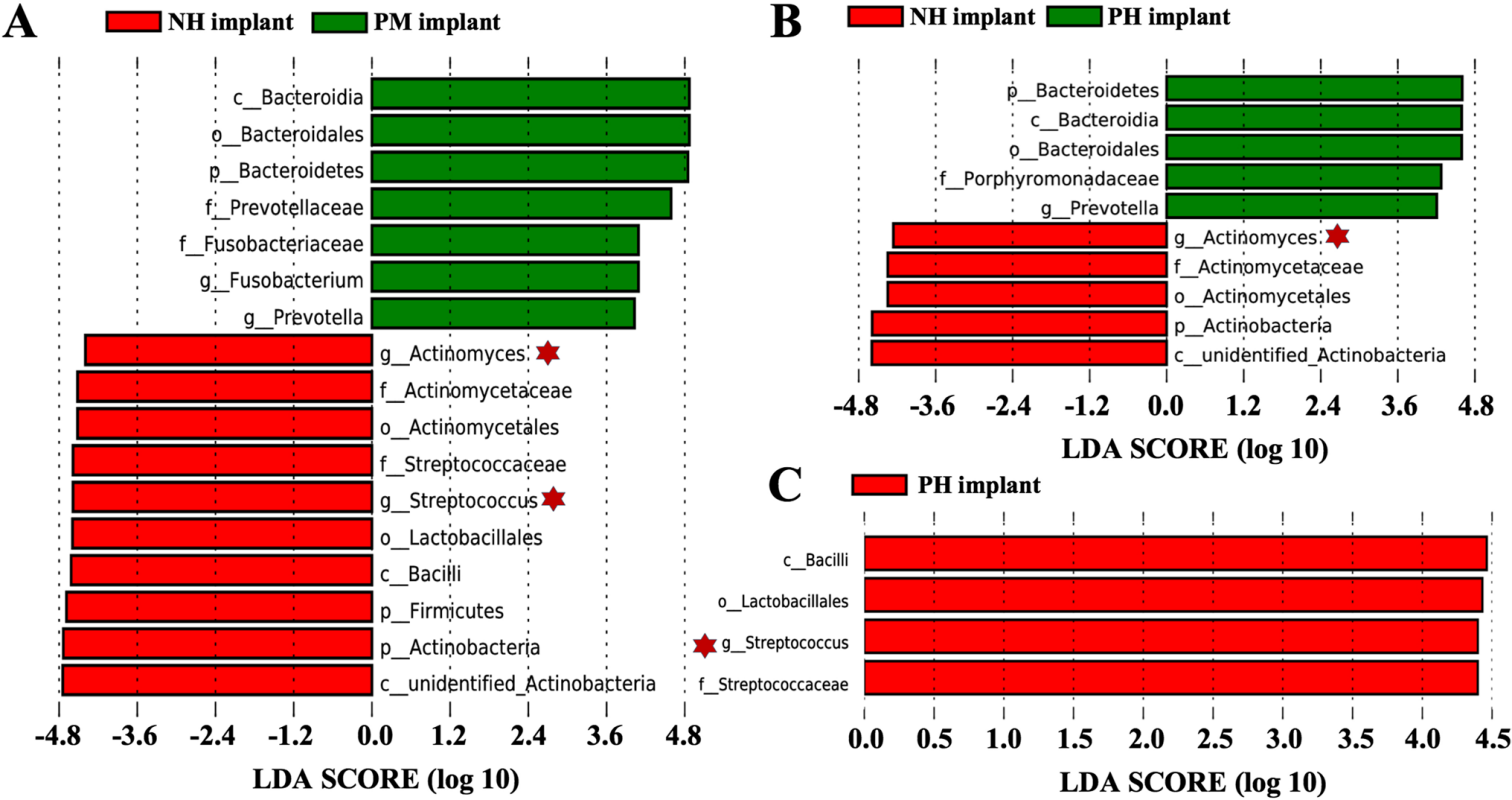
The distribution histograms of linear discriminant analysis (LDA) score comparing by LEfSe analysis. **A** NH implant and PM implant. **B** NH implant and PH implant. **C** PH implant and PM implant. Only taxa that were significantly higher than default in LDA scores (value = 4) were shown

To identify potential diagnostic biomarkers among groups, LEfSe was performed on a multilevel basis (Fig. 5). The LDA scores of the genera *Streptococcus* and *Actinomyces* were significantly higher, whereas those of *Prevotella* and *Fusobacterium* were remarkably lower in samples in the NH implant group (Fig. 5A) than in samples in the PM implant group (*P* < 0.05). Moreover, as shown in Fig. 5B, a lower abundance of the genus *Actinomyces* and a higher abundance of *Prevotella* were detected in the samples from the PH implant group than in the samples from the NH implant group (*P* < 0.05). However, the LEfSe results for samples in the PH implant and PM implant groups only showed that the genus *Streptococcus* was significantly higher in samples in the PH implant group (Fig. 5C).

### Bacterial composition of subgingival plaques between the teeth and implants

**Fig. 6:**
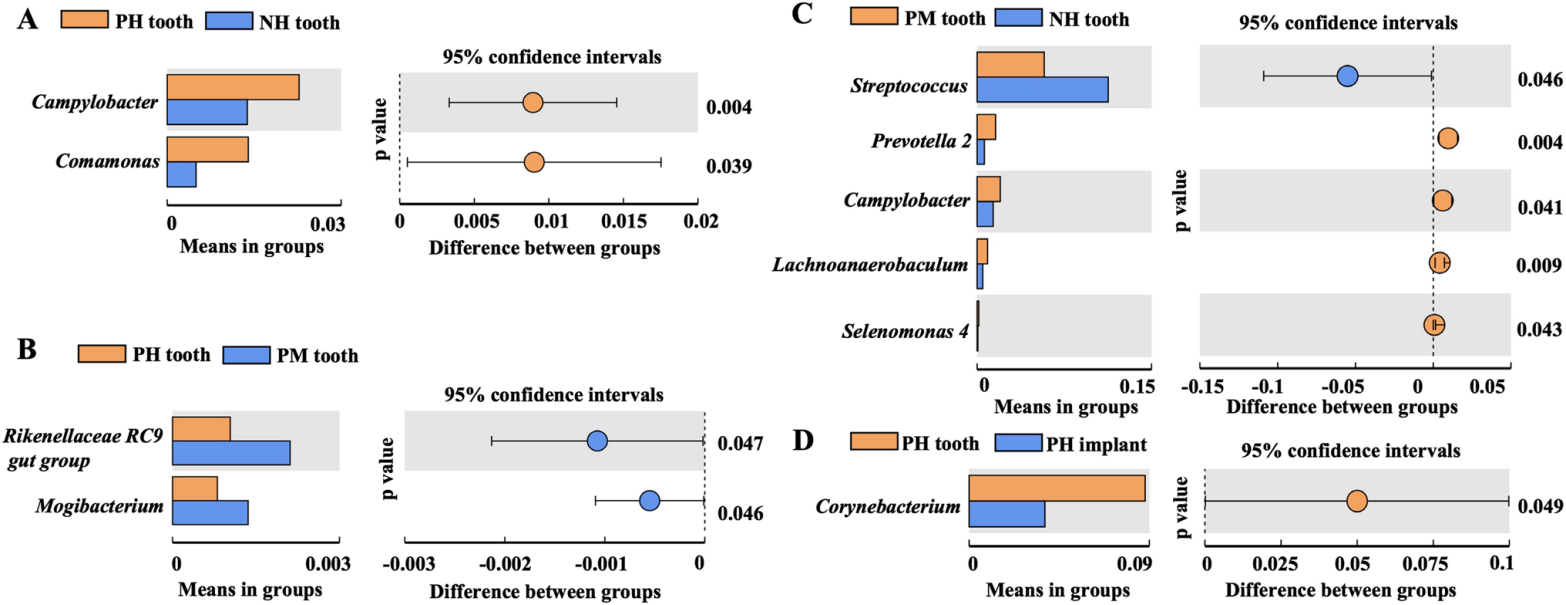
Relative microbial abundance of genus between the teeth and implants by Welch’s *t*-test. **A** Different genus between the PH tooth and NH tooth groups. **B** Different genus between the PH tooth and PM tooth groups. **C** Different genus between the PM tooth and NH tooth. **D** Different genus between the PH tooth and PH implant groups. Only taxa that were significantly different between groups were plotted (*P* < 0.05)

After comparing the subgingival and peri-implant plaques, we found that the genera *Campylobacter* and *Comamonas* were higher in samples in the PH tooth group than in samples in the NH tooth group (Fig. 6A). Samples in the PM tooth group expressed higher *Rikenellaceae RC9* and *Mogibacterium* than those in the PH tooth group (Fig. 6B). *Streptococcus* was higher while *Prevotella 2*, *Campylobacter*, and *Lachnoanaerobaculum* were lower in samples in the NH tooth group compared to the levels in samples in the PM tooth group (Fig. 6C). Moreover, the samples from periodontal and peri-implant sulci were compared, and *Corynebacterium* was the only differential genus with a significant difference between samples in the PH implant and PH tooth groups (Fig. 6D).

## Discussion

Healthy peri-implant tissues have become a major challenge in contemporary implant dentistry [30, 31]. Peri-implant health improves the life quality of patients [32]. There is strong evidence that peri-implant mucositis is caused by plaque, and is assumed to precede peri-implantitis [33]. Determining whether bacterial diversity of the peri-implant plaque could lead to peri-implant diseases is worth research. The present study compared the subgingival plaques associated with healthy and peri-implant mucositis implants, and evaluated whether the patient’s periodontal status was correlated with the peri-implant condition of implants. The human oral microbiome comprises hundreds of microorganisms, most of which exist in periodontal pockets and become subgingival dental plaques that are closely related to oral diseases, including peri-implant diseases [18]. The species composition of the periodontal and peri-implant microbiota varied widely among subjects [34].

The plaque index in the remaining teeth is one of the risk factors associated with peri-implant inflammatory disease, which means that patients with periodontitis are more susceptible to peri-implant mucositis [35]. Recently, a meta-analysis reported that implants placed in periodontitis patients were associated with a higher prevalence of implant loss and peri-implantitis than those placed in periodontally healthy patients [17]; thus, it is important to understand the changes in peri-implant bacterial structures between periodontally healthy and diseased individuals. In our study, we followed up with patients and compared the subgingival plaques of implants in the PH implant and NH implant groups, which could help us to understand the influence of periodontitis history on bacterial compositions. The results revealed that the abundances of the genera *Porphyromonas* and *Prevotella* were significantly higher in patients in the PH implant group, signifying that periodontitis patients had more gram-negative anaerobic bacteria around healthy implants, although the microbial structures in the NH implant and PH implant groups were not significantly different. A previous study reported that the plaques of peri-implantitis were mainly composed of gram-negative anaerobic bacteria [36]; thus, the accumulation of these bacteria might lead to higher susceptibility toward peri-implant diseases for periodontitis patients, and be considered a major risk factor for peri-implant diseases.

Dysbiosis of microorganisms was an important trigger for peri-implant mucositis, which was found to occur in more than 50% of all implant-carrying subjects. These microorganisms were also found to be responsible for a transitional and reversible phase in a patient’s progression from a healthy status to peri-implantitis, accompanied by the shift in bacterial communities from simple to complicated structures [37, 38]. Thus, subgingival plaques of patients with healthy implants and infected implants (PH implant and PM implant groups) were further analyzed in our study to investigate the changes in microbiome composition for the prevention and reversion of peri-implant mucositis. Our results showed that there were significant differences in the genera *Streptococcus*, *Granulicatella*, and *Rikenellaceae RC9* between patients in the PH implant and PM implant groups, and *Streptococcus* could also be selected as a potential biomarker through LEfSe analysis. However, there were no significant differences in general bacterial structures between patients in the PH implant and PM implant groups in our study, implying that peri-implant mucositis was an early stage of inflammation without remarkable changes in bacterial structures. It was reported that the bacterial composition in the deep peri-implant sulcus would be more complicated, and gram-negative anaerobes as well as opportunistic pathogens would be in the dominant position when peri-implantitis occurred [39]. In the present study, PM implants only had soft tissue inflammation without progressive marginal bone loss, so the bacterial structures were comparable to those of the PH implant group. However, classic periodontopathogens, including *Porphyromonas*, *Treponema*, and *Prevotella*, were detected in higher abundance in the PM implant group, similar to a previous study [40], illustrating that these genera might also be closely related to the development of peri-implant inflammation.

It was reported that microorganisms around the remaining teeth could translocate to the peri-implant sulcus [41]. Red clusters of periodontal pathogens have been detected in the peri-implant sulcus within 1 week after abutment connection for patients with periodontitis history [42]. Periodontopathogenic bacteria were only detected around implants in partially edentulous patients, while none of those bacteria were detected in completely edentulous patients [43]. In addition, the bacterial structures between peri-implant and periodontal sites in the same group of patients were similar, confirming that the bacterial composition tended to be uniform; only *Corynebacterium* was significantly different between the two sampling sites.

The complexity of the oral microbiome increases the difficulty of data analysis; thus, our results only showed differences at the genus level which was one of the limitations of this study. More advanced high-throughput sequencing technologies are needed for deep exploration in finding individual species for peri-implant disease. Then, in vitro experiments can help to confirm the mechanisms of these bacteria in the development of disease in the future. In addition, studies with more clinical samples are needed to determine the correlations between microbiota and clinical scores.

## Conclusions

In conclusion, the increased accumulation of *Fusobacterium* and *Prevotella* is associated with a higher risk of peri-implant mucositis. The peri-implant microbiota of periodontitis patients are more likely to be colonized with *Porphyromonas* and *Prevotella* than those driven from healthy individuals. Furthermore, the increase in periodontal pathogens and the decrease in health-associated bacteria in patients with periodontal disease may make them more likely to develop peri-implant diseases.

## Supplementary Information

Below is the link to the electronic supplementary material.

Supplementary file1 (DOCX 1216 KB)

## Data Availability

The data presented in this study are available on request from the corresponding author. The data are not publicly available due to ethical.
