# Supplementary material for: Microbiota analysis of peri-implant mucositis in patients with periodontitis history
Source: Clin Oral Investig. 2022 Jun 8;26(10):6223–33. doi: 10.1007/s00784-022-04571-1 (PMC9525361; doi:10.1007/s00784-022-04571-1)
Supplement: Supplementary file 1 — Supplementary file1 (DOCX 1216 KB) [file 784_2022_4571_MOESM1_ESM.docx]

Supplementary Information

Microbiota Analysis of Peri-implant Mucositis in Patients with Periodontitis History

Na Zhou^1†^, Haohao Huang^2†^, Hui Liu^1^, Qiang Li^1^, Guangwen Yang^1^, Yu Zhang^1^, Meng Ding^1^, Heng Dong^1, *^ and Yongbin Mou^1, *^


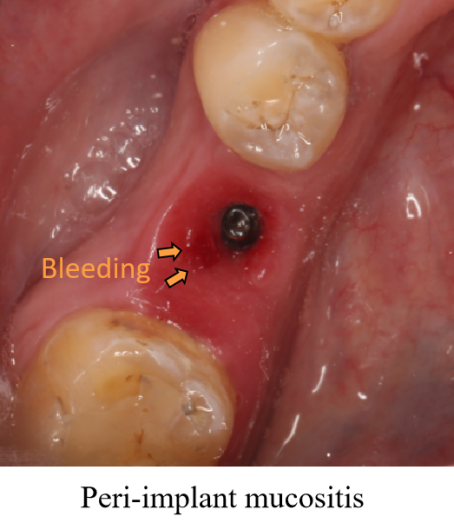


Figure S1. The representative image of peri-implant mucositis


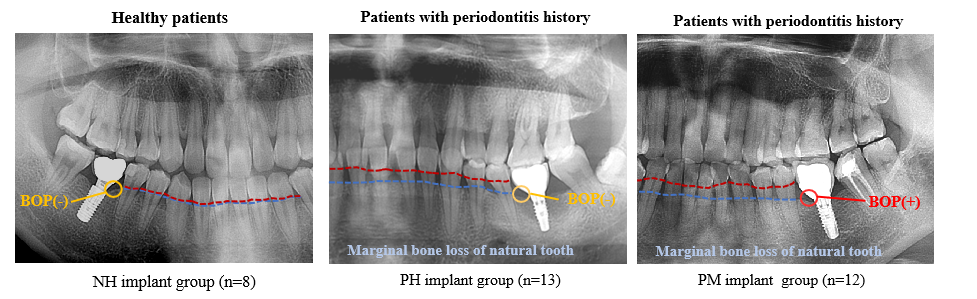


Figure S2. Representative X-ray images of patients in NH implant group, PH implant group and PM implant group.


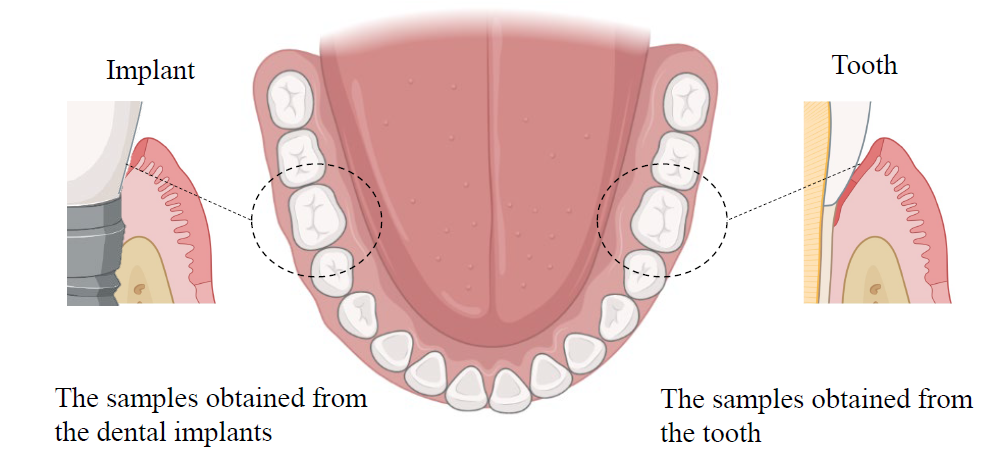


Figure S3. A schematic illustration of the collected plaque samples from the bottom of subgingival crevice


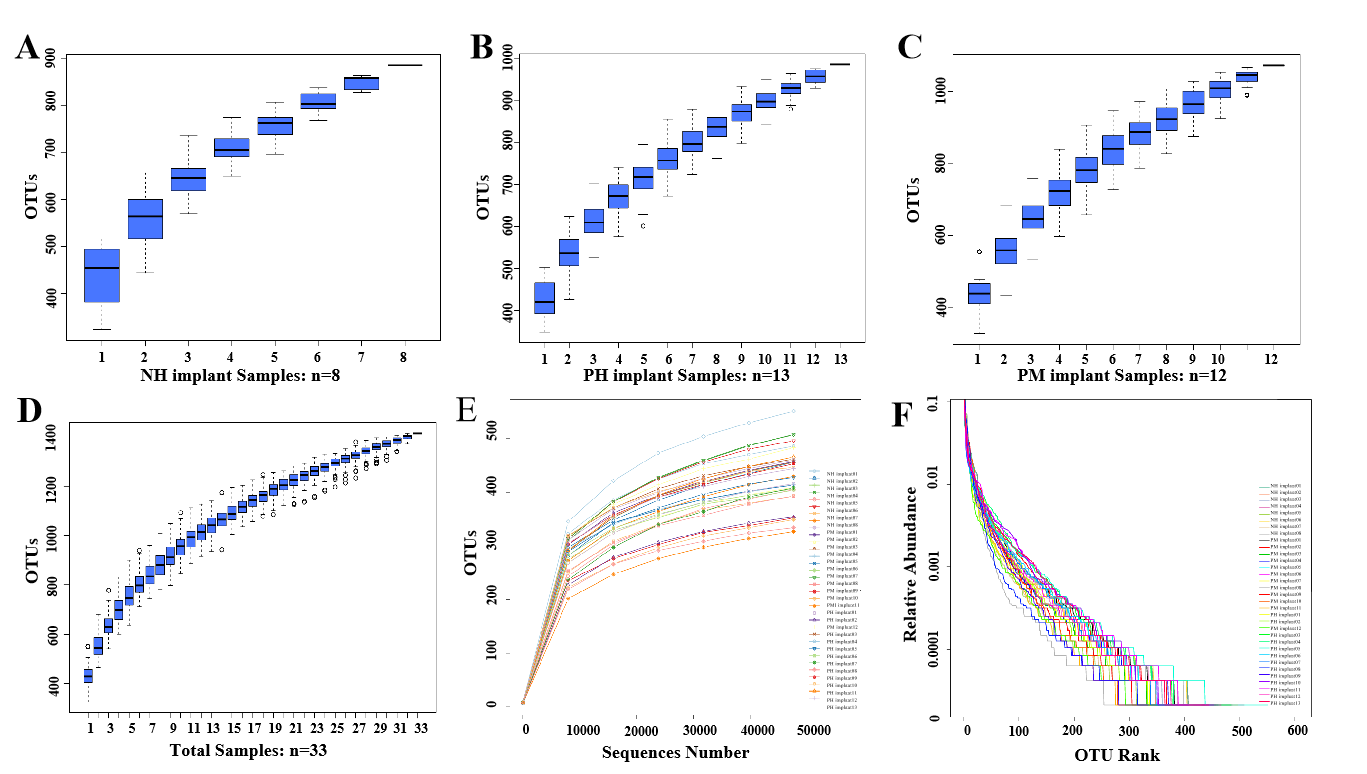


Figure S4. Species accumulation boxplots in (A) NH implant, (B) PH implant and (C) PM implant groups and (D) in all samples. The richness and evenness analysis of species. (E) Rarefaction Curve. (F) Rank abundance curve.

Table S1. Summary of samples, sequences and OTUs based on Illumina HiSeq platform

| Sample | Total Tag | Taxon Tag | OTUs (≥97% similarity) |
| --- | --- | --- | --- |
| NH implant01 | 69176 | 68610 | 574 |
| NH implant02 | 89849 | 89439 | 534 |
| NH implant03 | 77426 | 76913 | 519 |
| NH implant04 | 84845 | 84421 | 601 |
| NH implant05 | 89156 | 88770 | 488 |
| NH implant06 | 81215 | 80655 | 565 |
| NH implant07 | 65323 | 65113 | 461 |
| NH implant08 | 79596 | 79431 | 389 |
| PH implant01 | 73384 | 72738 | 532 |
| PH implant02 | 84457 | 83886 | 548 |
| PH implant03 | 77489 | 77148 | 481 |
| PH implant04 | 86797 | 86336 | 546 |
| PH implant05 | 83300 | 82696 | 473 |
| PH implant06 | 82131 | 81531 | 473 |
| PH implant07 | 82815 | 82261 | 449 |
| PH implant08 | 84820 | 84276 | 590 |
| PH implant09 | 91497 | 90983 | 459 |
| PH implant10 | 72274 | 72116 | 413 |
| PH implant11 | 84479 | 84263 | 404 |
| PH implant12 | 80844 | 79190 | 498 |
| PH implant13 | 73523 | 72277 | 475 |
| PM implant01 | 70122 | 68546 | 395 |
| PM implant02 | 91666 | 90852 | 571 |
| PM implant03 | 85010 | 84116 | 539 |
| PM implant04 | 76778 | 76190 | 643 |
| PM implant05 | 80945 | 80414 | 486 |
| PM implant06 | 75856 | 74866 | 463 |
| PM implant07 | 69000 | 68769 | 500 |
| PM implant08 | 69161 | 68928 | 392 |
| PM implant09 | 75908 | 75581 | 542 |
| PM implant10 | 76730 | 75272 | 512 |
| PM implant11 | 70846 | 69554 | 556 |
| PM implant12 | 74420 | 72426 | 518 |


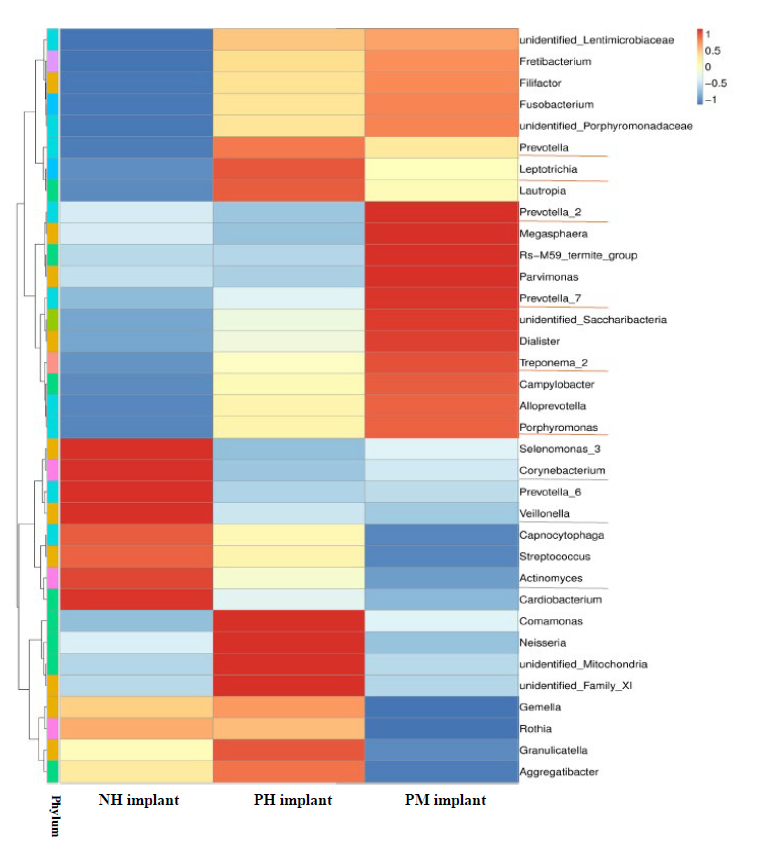


Figure S5. Cluster analysis in generic level for three groups. Each row represents one genus and each column represents one group. Orange lines mark the disease-related genera and gray lines mark the health-related genera.


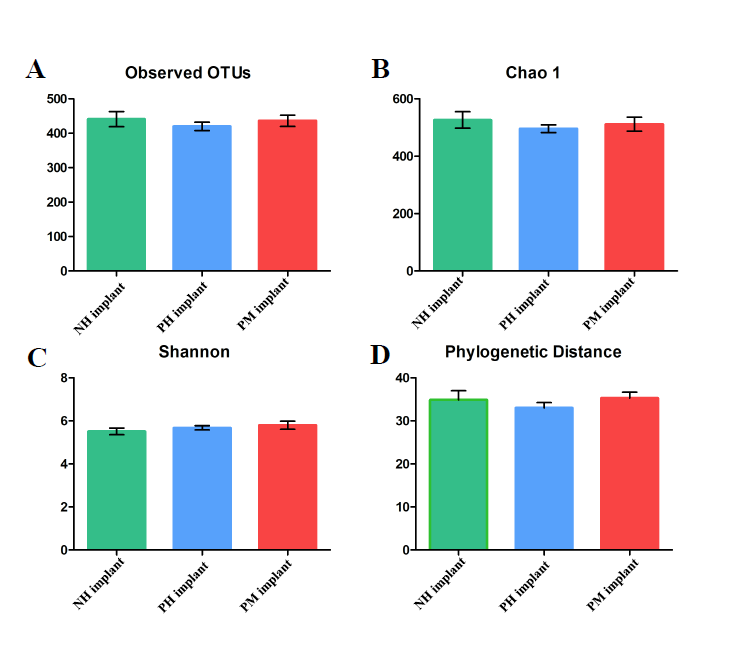


Figure S6. Analysis of microbial diversity in three groups. (A) Numbers of observed OTUs. (B) Analysis of species richness by Chao 1. (C) Analysis of microbial community diversity with Shannon index. (D) Measurement of community diversity with phylogenetic distance.
